# Case Report: Vancomycin-Induced Thrombocytopenia in a Burn Patient

**Published:** 2008-07-25

**Authors:** Ronald Pauldine, Aliaksei Pustavoitau

**Affiliations:** Department of Anesthesiology and Critical Care Medicine, Johns Hopkins Bayview Medical Center, Baltimore, MD; Department of Anesthesiology and Critical Care Medicine, Johns Hopkins Hospital, Baltimore, MD

## Abstract

**Objective:** Report an unusual cause of thrombocytopenia in a burn patient and provide a brief review of causes of thrombocytopenia in critically ill patients. **Methods:** Case report format and selected review of PubMed search including the search terms thrombocytopenia, critical illness, and vancomycin. **Results:** Reports of vancomycin-induced thrombocytopenia are uncommon. **Conclusion:** Drug-induced thrombocytopenia is a rare but important consideration in the evaluation of thrombocytopenia in critically ill patients. An increasing number of patients are treated with potentially causative agents including vancomycin and the diagnosis may be easily overlooked.

*Thrombocytopenia* is a relatively common finding in critically ill patients. Many potential causes are frequent and simultaneously present in any given patient and less common causes including drug-induced mechanisms can easily be overlooked. We present a case of a rarely encountered but important consideration in the differential diagnosis of thrombocytopenia.

## CASE REPORT

**Figure 1:**
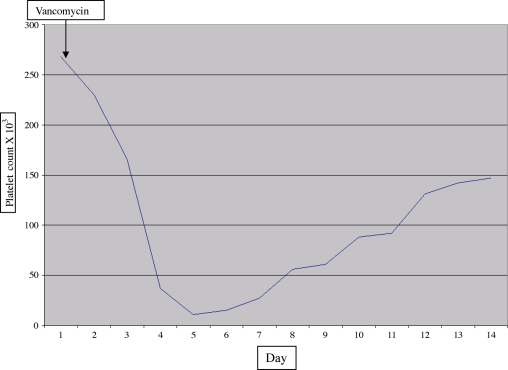
Platelet count following repeat treatment with vancomycin. Note the duration of significant thrombocytopenia in the setting of renal failure.

A 60-year-old gentleman with a past medical history of essential hypertension, dyslipidemia, and stable coronary artery disease was admitted with steam burns to 40% of total body surface area involving his abdomen, bilateral lower extremities, and bilateral upper extremities. He had been working as a pipe fitter attempting to repair underground steam pipes when the mishap occurred. Medications at the time of admission included aspirin, hydrochlorothiazide, and atorvastatin. He had no known allergies to medications. His burn wounds were successfully excised and grafted with good results. However, his hospital course was complicated by acute renal failure requiring dialysis and ventilator-associated pneumonia with respiratory failure leading to tracheostomy and prolonged ventilator dependence. His pneumonia was treated initially with intravenous vancomycin and piperacillin/tazobactam. Methicillin-resistant *Staphylococcus aureus* was isolated from the sputum and antibiotic coverage was tailored with discontinuation of piperacillin/tazobactam. Treatment with vancomycin was continued for 14 days. Several days after completion of antibiotic therapy the patient continued to have clinical signs of infection including elevated white blood cell count, fever, increased sputum production, lack of improvement in arterial to inspired oxygen ratio, and a persistent, albeit subtle infiltrate on chest radiograph. Prior sputum cultures had also demonstrated the presence of a resistant *Acinetobacter* species known to be an occasional colonizing organism in our intensive care unit. Failed treatment of ventilator-associated pneumonia and tracheobronchitis were considered as possible explanations for the clinical picture. Fiberoptic bronchoscopy with bronchoalveolar lavage was performed. Initial gram stain of the lavage fluid revealed gram-positive cocci, and vancomycin was restarted. The following day, vancomycin was discontinued and therapy with linezolid was initiated. At the time vancomycin was restarted the platelet count was 268,000/mm^3^. Over the next 3 days the platelet count dropped precipitously to a nadir of 10,000/mm^3^. On examination, mild, spontaneous bleeding was evident around the dialysis catheter and oozing was present from several healing wounds. Laboratory analysis results included prothrombin time 12.9 seconds, international normalized ratio 1.3; partial tissue thromboplastin time 30.1 seconds, ratio 1.0; fibrinogen 483 mg/dL and fibrin degradation products less than 10 μg/ml. An assay for heparin-induced antibodies was negative. Given the clinical scenario and evidence of spontaneous bleeding, platelet transfusion was attempted with minimal hematologic and clinical response. A diagnosis of drug-induced thrombocytopenia was considered as was idiopathic thrombocytopenic purpura. Bronchoalveolar lavage fluid grew diagnostic quantities of *Staphylococcus aureus* sensitive to macrolide antibiotics and therapy was continued with azithromycin. Therapy with corticosteroids was initiated without improvement. The platelet count eventually recovered to levels above 50,000/mm^3^ after 10 days while patient was continued on dialysis for acute renal failure (Fig 1). A careful review of possible offending agents led to vancomycin as a presumptive cause of thrombocytopenia. Testing for vancomycin-dependent antiplatelet antibodies was requested and revealed the presence of IgG vancomycin-dependent, platelet-reactive antibodies. These finding are consistent with a diagnosis of vancomycin-induced immune thrombocytopenia.^1^

## DISCUSSION

*Thrombocytopenia* defined as a platelet count less than 100,000/mm^3^, is a relatively common finding occurring in up to 35% of patients in the surgical intensive care units.^2^ Thrombocytopenia can occur as the result of impaired platelet production or increased consumption. Consumption may be related to both nonimmunologic and immunologic mechanisms. The differential diagnosis of thrombocytopenia in the intensive care unit is summarized in Table 1. The most frequent causes in this population are sepsis and disseminated intravascular coagulation accounting for more than 75% of all cases. Other causes of thrombocytopenia in the intensive care unit include dilutional thrombocytopenia from massive blood loss (8%); thrombotic microangiopathy (thrombotic thrombocytopenic purpura, hemolytic-uremic syndrome, severe malignant hypertension, and chemotherapy-induced microangiopathy) (1%); heparin-induced thrombocytopenia (1%); immune thrombocytopenia (3%); and drug-induced thrombocytopenia (10%).^3^ Drug-induced thrombocytopenia can occur due to decreased platelet production, that is, bone marrow toxicity or due to an immune-mediated increase in platelet destruction.^4^ Aster has recently reviewed the mechanisms of immune-mediated platelet destruction.^5^,^6^ Mechanisms of immune-mediated platelet destruction include interactions of hapten-dependent antibodies as well as drug-specific antibodies, drug-induced autoantibodies, and immune complex-mediated pathways. Recovery of the platelet count is anticipated after withdrawal of the offending agent.

Vancomycin is frequently employed in the treatment of gram-positive bacterial infections and has become a mainstay in the treatment of serious infections in hospitalized patients. Reports implicating vancomycin as a causal agent of thrombocytopenia are rare.^7^,^8^ Von Drygalski and others recently reported a series of patients with thrombocytopenia, who had been referred for testing of vancomycin-dependent, platelet-reactive antibodies because of clinical suspicion of vancomycin-induced thrombocytopenia.^1^ Although the incidence of vancomycin-induced thrombocytopenia remains unknown, this study highlights the possibility of serious bleeding complications in patients receiving antimicrobial therapy with vancomycin and underscores the importance of considering this cause of thrombocytopenia in the intensive care unit. The diagnosis of drug-induced thrombocytopenia is often difficult in intensive care unit patients. Frequently, there is more than 1 potential cause for a low platelet count and a number of potential causative agents may have been administered. In this case, the patient received a short course of linezolid before antibiotic therapy was de-escalated based on culture results. Although linezolid is known to be associated with thrombocytopenia, the majorities of cases reported are dependent on duration of therapy and are easily reversible on discontinuing the drug.^9^ Arriving at the correct diagnosis is based on ruling out common causes and reviewing the timing of onset of thrombocytopenia with potentially offending agents. George has reviewed the evidence for and serially updated agents associated with drug-induced thrombocytopenia.^10^–^12^ The peripheral blood smear can be useful in evaluating for the presence of microangiopathic processes. Bone marrow studies may be useful in selected patients. Serology may be important where assays are available for specific antibodies. Care must be taken, however, in interpretation of antiplatelet antibodies alone when heparin-induced thrombocytopenia is considered. In this, patient laboratory testing and the clinical scenario ruled out the most frequent causes of thrombocytopenia in the critically ill. Failure to achieve a sustained increase in platelet count after platelet transfusion was consistent with platelet destruction as the primary mechanism. Heparin-induced thrombocytopenia (HIT) has been extensively reviewed in the literature and is a frequent consideration in hospitalized patients with low platelet counts. In this instance heparin-induced mechanisms were considered but rejected based on the clinical picture and subsequent negative serology. Although HIT is a major concern in a critically ill patient due to almost universal exposure to the drug, it is diagnosed very infrequently.^13^,^14^ A simple model named the “4 T's” system was created and validated to assess the pretest probability of a diagnosis of HIT.^15^ The 4 Ts include degree of thrombocytopenia, timing of clinical sequelae, thrombosis in vascular beds, and other possible explanations for thrombocytopenia. On the basis of this model, the patient had a low-to-intermediate probability of HIT on clinical grounds and with further laboratory assays negative for antibodies, HIT was very unlikely.^16^

Further search for possible causative agents placed vancomycin at the top of the list. This was later confirmed by the demonstration of vancomycin-dependent antiplatelet antibodies. The delayed recovery after discontinuation of vancomycin is consistent with delayed drug clearance due to renal failure and was observed in the von Drygalski study.^1^ This report highlights the importance of maintaining an index of suspicion for drug-induced mechanisms as a cause for thrombocytopenia in patients treated with vancomycin. This mechanism may be overlooked in the critically ill burn patient because thrombocytopenia can often be attributed to multiple other causes.

## Figures and Tables

**Table 1:** Causes of thrombocytopenia in the intensive care unit

Sepsis
Disseminated Intravascular Coagulation
Massive Blood Loss
Drug-induced Thrombocytopenia
Immune Thrombocytopenia
Thrombotic Microangiopathy
Heparin-induced Thrombocytopenia
